# Cisplatin induced neurotoxicity is mediated by Sarm1 and calpain activation

**DOI:** 10.1038/s41598-020-78896-w

**Published:** 2020-12-14

**Authors:** Aysel Cetinkaya-Fisgin, Xinghua Luan, Nicole Reed, Ye Eun Jeong, Byoung Chol Oh, Ahmet Hoke

**Affiliations:** Department of Neurology, Johns Hopkins School of Medicine, 855 N. Wolfe St., Baltimore, MD 21205 USA

**Keywords:** Peripheral nervous system, Regeneration and repair in the nervous system

## Abstract

Cisplatin is a commonly used chemotherapy agent with significant dose-limiting neurotoxicity resulting in peripheral neuropathy. Although it is postulated that formation of DNA-platinum adducts is responsible for both its cytotoxicity in cancer cells and side effects in neurons, downstream mechanisms that lead to distal axonal degeneration are unknown. Here we show that activation of calpains is required for both neurotoxicity and formation of DNA-platinum adduct formation in neurons but not in cancer cells. Furthermore, we show that neurotoxicity of cisplatin requires activation of Sarm1, a key regulator of Wallerian degeneration, as mice lacking the Sarm1 gene do not develop peripheral neuropathy as evaluated by both behavioral or pathological measures. These findings indicate that Sarm1 and/or specific calpain inhibitors could be developed to prevent cisplatin induced peripheral neuropathy.

## Introduction

Cisplatin (CDDP) is a chemotherapy drug commonly used in systemic treatment of germ cell tumors, including cancers of the ovaries, testes, and solid tumors of the head and neck^1^. CDDP act as an antitumor agent by inducing inter- and intra-strand crosslinks in DNA and formation of DNA-platinum adducts which, results in apoptotic cell death of cancer cells^2^. A common dose-limiting side effect of systemic treatment with CDDP is chemotherapy-induced peripheral neuropathy (CIPN), a debilitating and painful condition that may hamper optimal anti-cancer treatment^3–5^. Peripheral neurotoxicity develops in approximately 50% of patients receiving CDDP^6^. Once established, there is no effective therapy and treatment is directed at symptomatic control of pain^7^.

Peripheral neuropathy due to neurotoxicity of CDDP is characterized by distal axonal degeneration, a condition similar to other axonal peripheral neuropathies. The exact molecular mechanisms that lead to distal axonal degeneration are not fully understood. However, a number of pathophysiological mechanisms have been proposed to explain this phenomenon. A prevailing hypothesis suggests that CDDP kills malignant cells and damages peripheral neurons by means of a similar mechanism of accumulation DNA-platinum adducts, which leads to apoptosis in cancer cells and dorsal root ganglion (DRG) neurons^8^. Other hypotheses that have been put forward include mitochondrial dysfunction, either by DNA crosslinking or oxidative stress^9,10^.

Although it has not been examined specifically in CDDP induced neurotoxicity, several proteases are involved in apoptosis and axonal degeneration in DRG neurons. For example, calpain inhibition has been shown to prevent paclitaxel-induced sensory neuropathy in mice^11^ and caspase 3 activation is required for distal axonal degeneration in a model of a toxic neuropathy induced by HIV envelop protein gp120^12^ as well as paclitaxel^13^. Furthermore, calpains mediate the breakdown of axonal cytoskeleton and degeneration of traumatically-injured axons depends on calpain activation as it can be delayed by expression of the endogenous calpain inhibitor, calpastatin^14^. In traumatic axonal injury, a key regulator of distal axonal degeneration is Sarm1 (sterile alpha and armadillo motif1). Mice lacking the Sarm1 gene are resistant to Wallerian degeneration^15,16^, and neuropathy caused by vincristine^17^, paclitaxel and metabolic syndrome^18^.

In this study we examined the role of several proteases and Sarm1 in CDDP-induced neurotoxicity and show that accumulation of DNA-platinum adducts in DRG neurons and distal axonal degeneration are dependent on calpain activation and development of neuropathy in mice requires Sarm1.

## Results

### Dose-dependent neurotoxicity of CDDP

**Figure 1:**
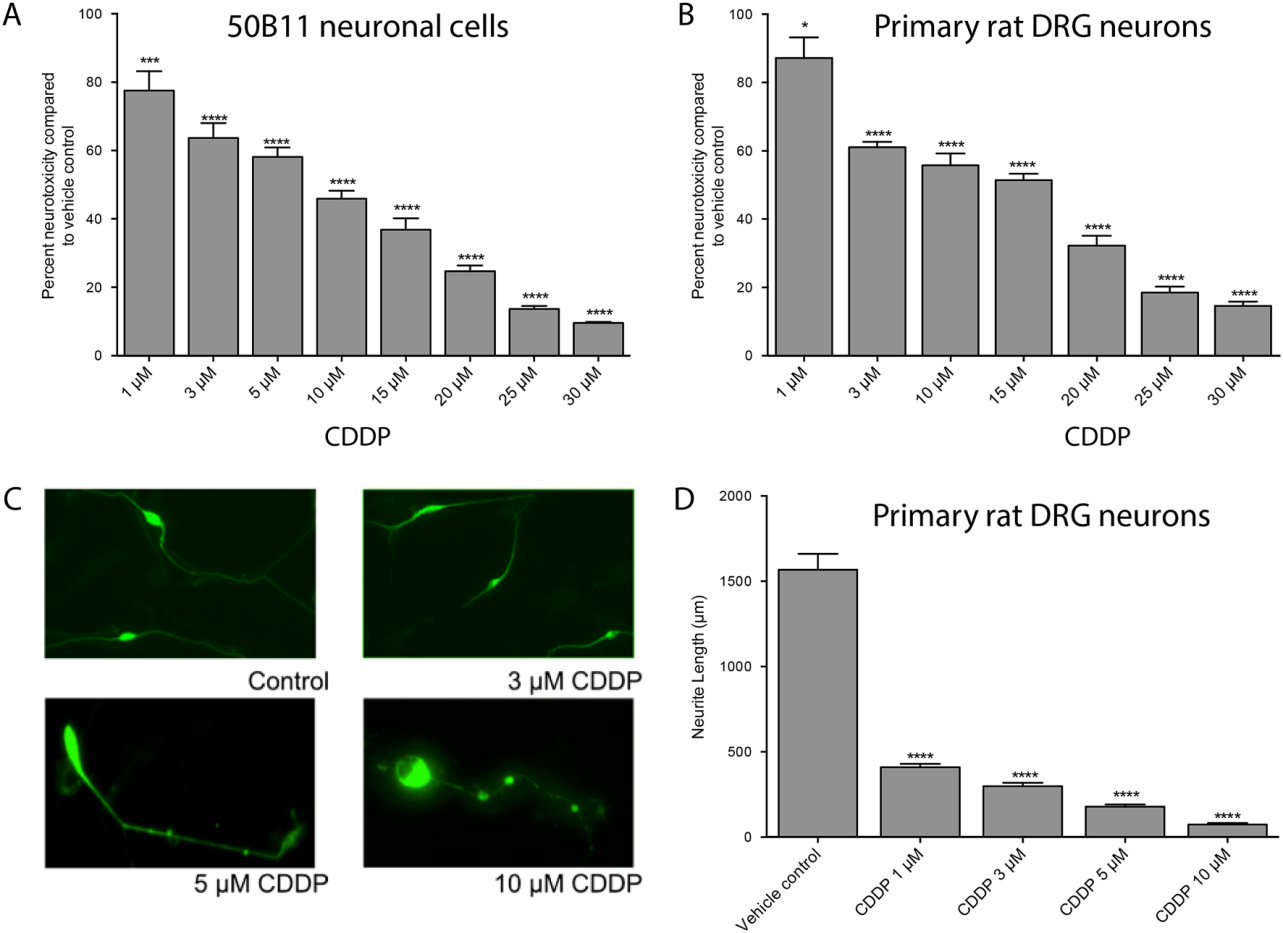
Cisplatin caused a dose dependent toxicity on rat immortalized DRG neuronal cell line (**A**) and primary rat DRG neurons (**B**) as assessed by ATP measurements. Exposure of primary rat DRG neurons also caused a dose-dependent degeneration of neurites (**C**,**D**). Panel **C** shows degeneration and blebbing in representative images of fixed and stained DRG neurons. (n = 3/condition, one-way ANOVA, *p < 0.05, ***p < 0.0005, ****p < 0.0001, error bars denote SEM).

We examined the neurotoxicity of CDDP in the rat DRG neuronal cell line and in primary rat DRG neurons. As seen in Fig. 1A,B, there is a dose-dependent reduction in cellular ATP levels in both neuronal cell types with EC_50_ at 10–15 µM. However, significant neurotoxicity can be appreciated even at lower doses when one examines axonal blebbing and reduction in neurite length in primary rat DRG neurons (Fig. 1C,D).

### CDDP activates Calpain and Caspase 3/7

**Figure 2:**
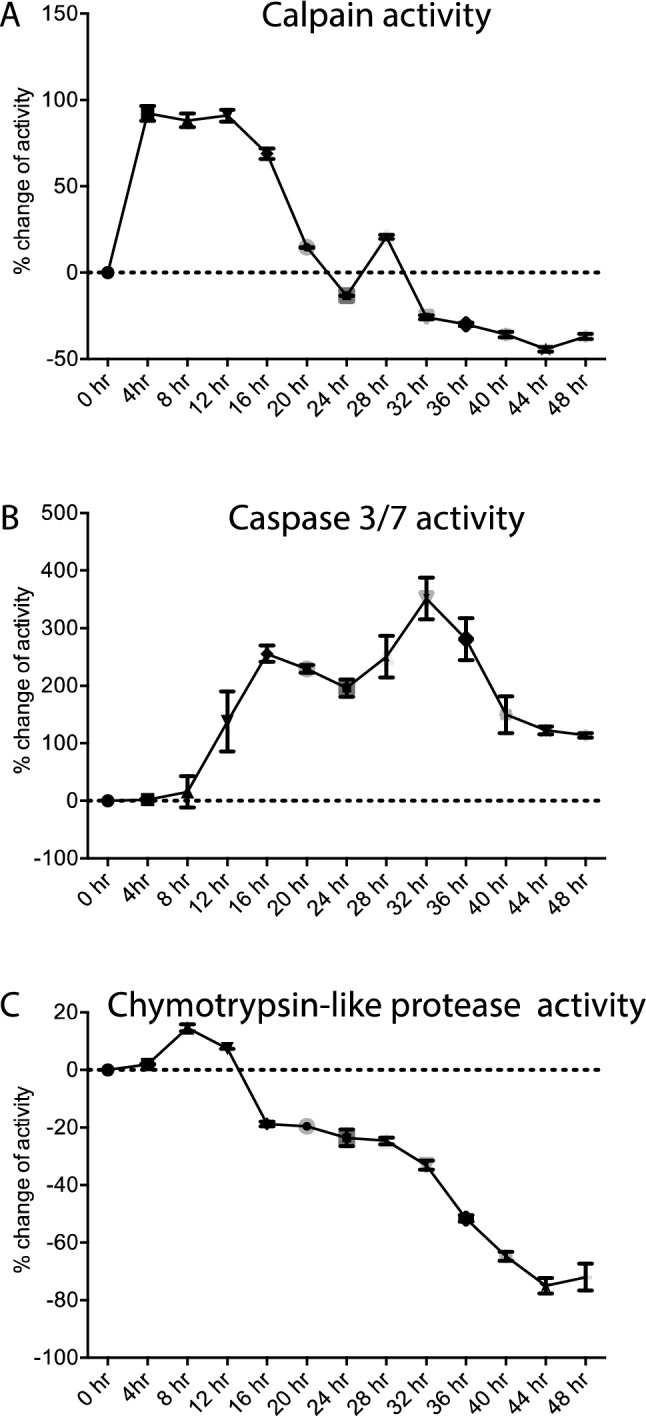
Calpain (**A**), Caspase 3/7 (**B**) and Chymotrypsin-like Protease (**C**) activity in 50B11 DRG neuronal cells exposed to CDDP (3 µM). Differentiated 50B11 DRG neuronal cells were exposed to 3 µM CDDP and Calpain, Caspase 3/7 and chymotrypsin-like protease activity assays were done every 4 h and normalized to protein concentration. Calpain activation starts within the first 4 h of CDDP addition to wells, followed by caspase 3/7 activation around 8 h and proteasome inhibition start at 16th hour (n = 4/condition, one-way ANOVA, ****p < 0.0001, error bars denote SEM).

As seen in Fig. 2, addition of 3 µM of CDDP to differentiated rat DRG neuronal cell line results in rapid increase in calpain activity within the first 4 h followed by reduction starting at 12 h and going below the control cultures by 32 h. In contrast, Caspase 3/7 activity is not increased until after 12 h and is sustained throughout the 48 h examined. Chymotrypsin-like proteasome activity did not show any significant increase but was reduced starting at 16 h. The activation or inhibition of all three proteases was proportional to the CDDP concentration (Supplementary Fig. 1). Since, ubiquination is an ATP dependent process, inhibition of the UPS may be related to the ATP depletion during axonal degeneration and a secondary phenomenon. Therefore, we decided to focus on the relationship between caspase and calpain activities.

**Figure 3:**
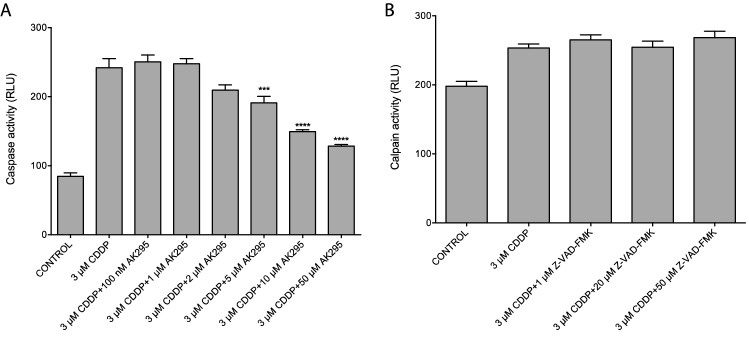
Calpain inhibitor AK295, inhibited CDDP-induced caspase 3/7 activation (**A**) suggesting that caspase activity is downstream of calpain activation (RLU: relative light units). In contrast, pan-caspase inhibitor Z-VAD-FMK did not have any effect on calpain activity (**B**) (n = 6, **p < 0.01, ****p < 0.0001, one way ANOVA with multiple comparisons, error bars denote SD).

In order to examine the relationship between caspase and calpain activities, we exposed differentiated rat DRG neuronal cell line to 3 µM CDDP with and without inhibitors and measured calpain and caspase activities. As seen in Fig. 3, a pan-caspase inhibitor was unable to influence the activation of calpain, but a specific calpain inhibitor, AK295 was able to prevent increase in caspase activity in a dose-dependent manner. The specificity of the assays to measure the activation of the proteases were tested with inhibitors (Supplementary Fig. 2).

**Figure 4:**
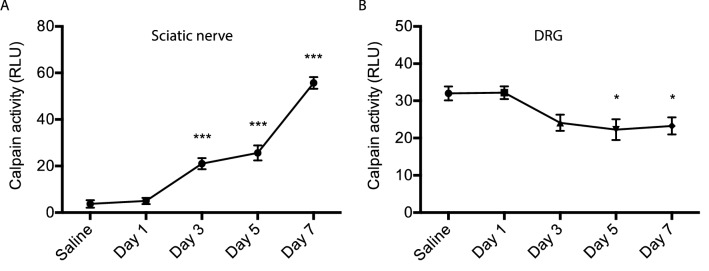
In-vivo time course of calpain activation in mice treated with CDDP. Calpain activation starts 3 days after CDDP in sciatic nerves and continues until 7 days post-injection (**A**). In contrast, there was no significant activation of calpain in DRG samples (**B**) (n = 5 per group, *p < 0.05, ***p < 0.005, one way ANOVA with multiple comparisons, error bars denote SD).

To examine whether calpain activation occurs in vivo, we administered CDDP to adult wild type mice and measured calpain activity in sciatic nerves and DRGs. As seen in Fig. 4, increase in calpain activity was local to the axons and seen in sciatic nerves but not in sensory neuronal cell bodies in the DRG. Time course was slower than in vitro with increased activity being observed up to 7 days after CDDP injection.

### Calpain activation is required for formation of DNA-platinum adducts in neurons

**Figure 5:**
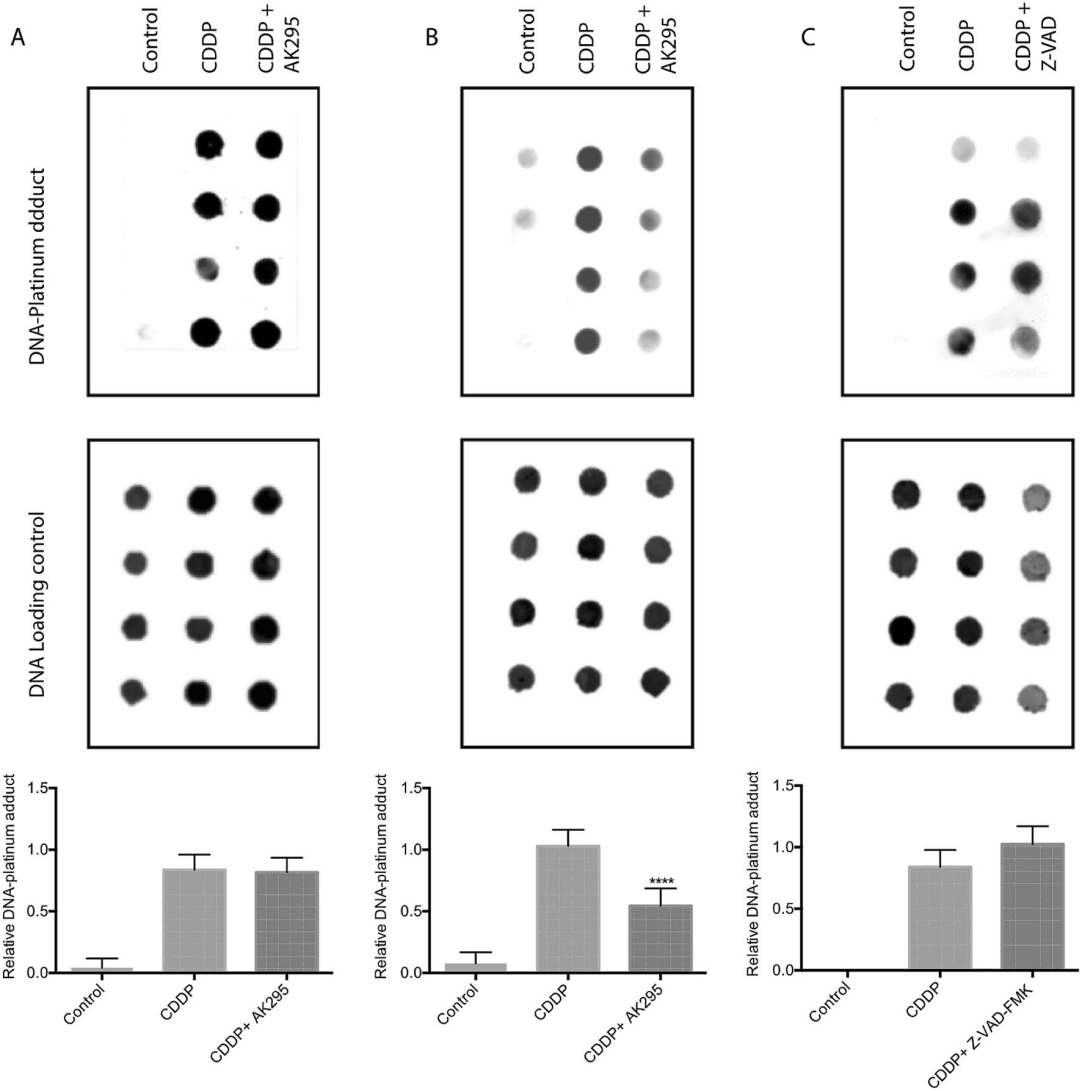
Formation of DNA-platinum adducts by CDDP was not blocked by calpain inhibitor AK-295 in cancer cells (**A**), but it was blocked in neuronal cells (**B**). Caspase inhibition by ZVAD had no effect on DNA-platinum adduct formation in neuronal cells (**C**). Uncropped dot blots show representative blots (n = 4) for each condition and quantitation is shown in graphs below each (n = 8 per group, ****p < 0.001, one-way ANOVA with multiple comparisons, error bars denote SD).

The primary mechanism by which CDDP causes neurotoxicity is by formation of DNA-platinum adducts. Since we observed a very rapid increase in calpain activity upon exposure to CDDP, we asked if DNA-platinum adduct formation is dependent on calpain activation. In PA-1 cancer cells, inhibition of calpain activity by AK295 did not affect formation of DNA-platinum adduct formation (Fig. 5A). Similar results were obtained with other cancer cell lines and with two different types of calpain inhibitors, AK295 and MDL28170 (Supplementary Fig. 3).

**Figure 6:**
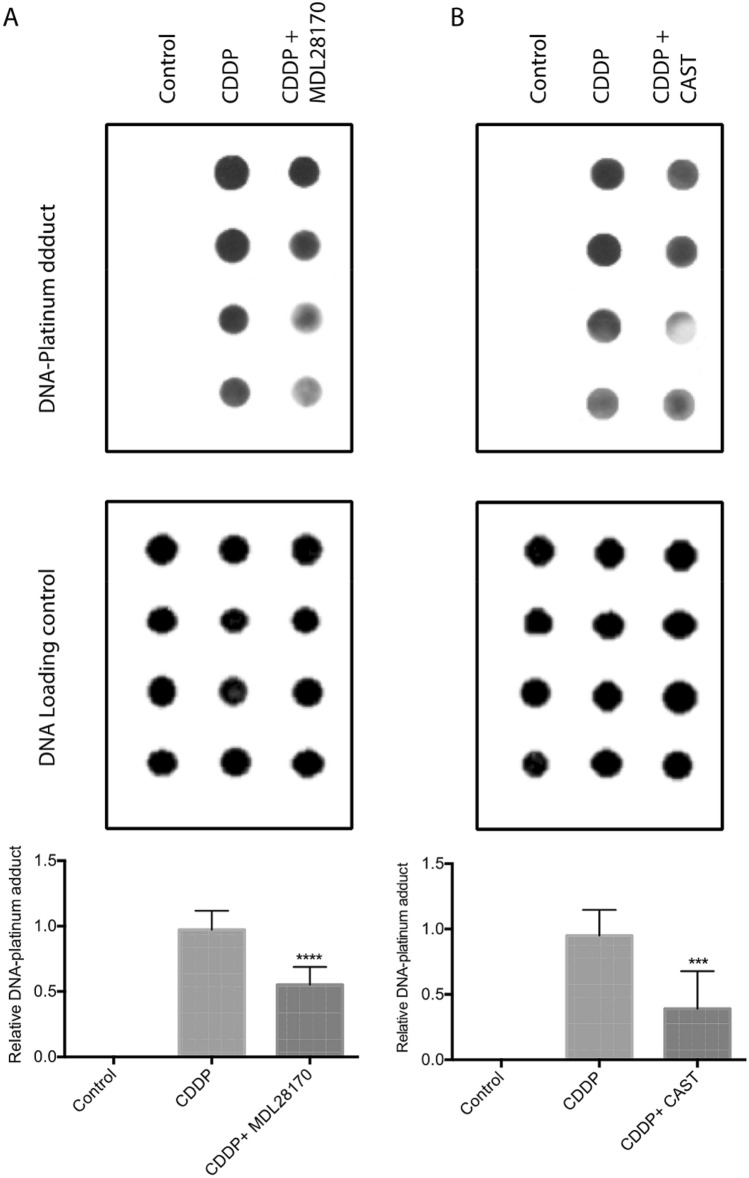
Formation of DNA-platinum adducts by CDDP in neuronal cells was blocked by two additional calpain inhibitors MDL28170 (**A**) and calpastatin (**B**). Uncropped dot blots show representative blots (n = 4) for each condition and quantitation is shown in graphs below each (n = 8 per group, ***p < 0.005, ****p < 0.001, one-way ANOVA with multiple comparisons, error bars denote SD).

In contrast, calpain inhibitor, AK295 partially blocked formation of DNA-platinum adducts in CDDP-treated neuronal cells (Fig. 5B). To confirm the specificity of AK295, we examined the effects of other calpain inhibitors MDL28170 and calpastatin (CAST) and saw a similar pattern of partial inhibition (Fig. 6A,B). In contrast the pan-caspase inhibitor ZVAD-FMK did not have any effect on formation of DNA-platinum adducts in CDDP-treated neuronal cell line (Fig. 5C).

### Genetic deletion of Sarm1 prevents development of CDDP-induced neuropathy

**Figure 7:**
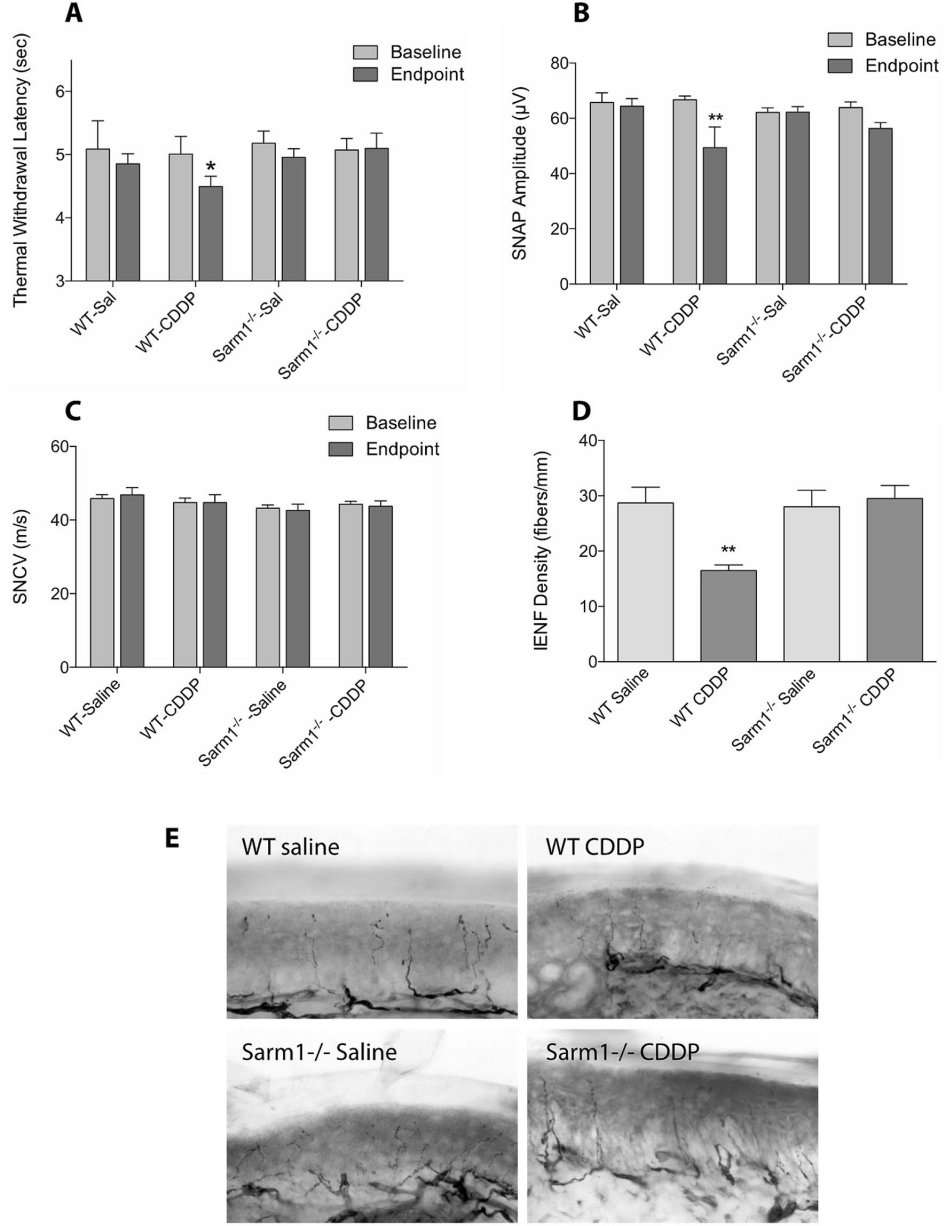
CDDP induced peripheral neuropathy in Sarm 1^−/−^ mice. Although WT mice given CDDP developed peripheral neuropathy as assessed by thermal withdrawal latency (**A**), sensory nerve action potential (**B**) and changes in epidermal innervation (**D**,**E**), Sarm 1^−/−^ mice did not develop peripheral neuropathy. There was no change in sensory nerve conduction velocity in WT or Sarm 1^−/−^ mice (**C**) (n = 5 per WT mice and n = 7 per Sarm 1^−/−^ mice, *p < 0.05, **p < 0.005, one-way ANOVA with multiple comparisons, error bars denote SD).

Treatment of wild type mice with CDDP (4 mg/kg/week) for 4 weeks resulted in development of a sensory neuropathy characterized by thermal hyperalgesia, and reduction in epidermal nerve fibers and amplitude of sensory evoked responses (Fig. 7). In contrast, Sarm1^−/−^ mice did not develop thermal hyperalgesia or reduction in epidermal nerve fiber density or amplitude of evoked sensory responses. As CDDP causes a primarily axonal neuropathy, there was no change in sensory conduction velocities.

**Figure 8:**
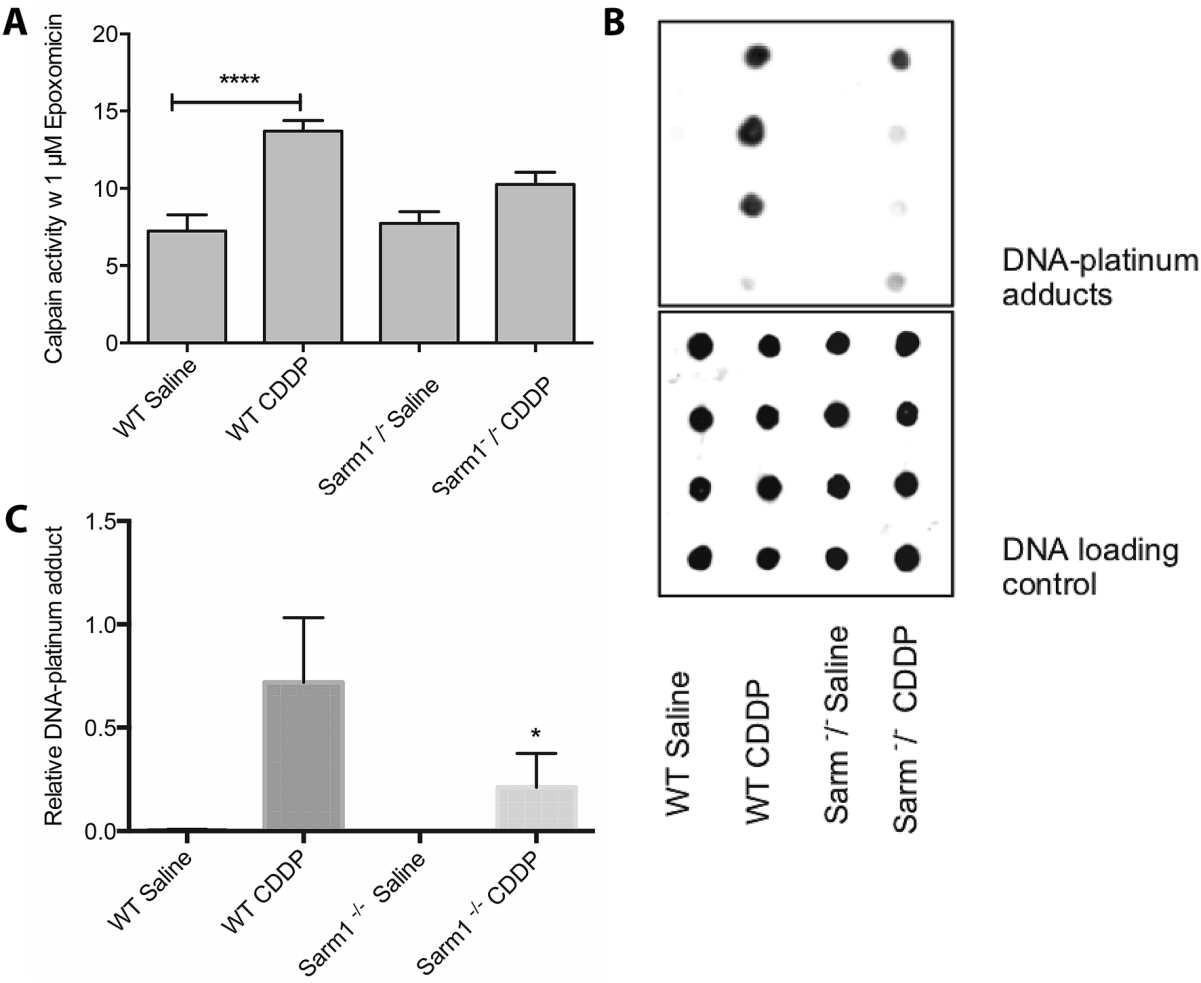
Calpain activation (**A**) and DNA-platinum adduct formation (**B**,**C**) in WT and Sarm1^−/−^ mice treated with CDDP (Images in B are uncropped dot blots of representative 4 samples from each group; quantitation in panels A and C are from n = 5 per WT mice and n = 7 per Sarm 1^−/−^ mice, *p < 0.05, ****p < 0.001, one-way ANOVA with multiple comparisons, error bars denote SD).

Since Sarm1 activation is required for degeneration of distal axons upon treatment with CDDP, we examined the relationship between calpain activation, DNA-platinum adduct formation and Sarm1 in the Sarm1^−/−^ mice. As seen in Fig. 8A, CDDP-treated Sarm1^−/−^ mice did not exhibit an increase in calpain activity in the sciatic nerves. Same animals also had only a minimal increase in DNA-platinum adducts in the DRG compared to the wild type mice, which exhibited a marked increase (Fig. 8B,C).

## Discussion

Sensory neurotoxicity is a major, dose-limiting side effect of cisplatin, and recovery from CDDP-induced neuropathy is often incomplete; persisting in up to 55% of patients, even years after the cessation of treatment^19^. CDDP-induced peripheral neuropathy is characterized by distal-to-proximal axonal degeneration. Although the exact mechanisms of axonal degeneration are still being examined, it likely utilizes molecular machinery common to what is in Wallerian degeneration. This assumption is based on several recent advances that showed that a key player of Wallerian degeneration, Sarm1, is also important in slow onset distal axonal degeneration due to several other chemotherapy drugs and metabolic syndrome^17,18^.

Similar to the observations in vincristine neuropathy^17^, paclitaxel neuropathy and high fat diet-induced neuropathy^18^, Sarm1^−/−^ mice were resistant to development of CDDP-induced distal axonal sensory neuropathy as evidenced by lack of development of thermal hyperalgesia, reduction in sensory evoked response amplitude or epidermal innervation of the hind limb. The mouse model of CIPN that we used in this study faithfully replicates the early stages of peripheral neuropathy seen in patients where they exhibit painful distal paresthesias before they develop significant large fiber dysfunction such as balance issues or muscle weakness due to motor denervation. This, and other studies now confirm the key role Sarm1 plays in mediating axonal degeneration from multiple toxic and metabolic causes.

It has been postulated that Sarm1 activation is required for calpain activation which is thought to be one of the most immediate steps in Wallerian degeneration before dissolution of cytoskeleton stakes place^20^. Hence, we were expecting calpain activation to be a late event during CDDP-induced neurotoxicity. However, we were surprised to find the relatively rapid calpain activation in CDDP treated neuronal cultures. Furthermore, this calpain activation was a prerequisite for caspase activation and formation of DNA-platinum adducts. We don’t believe this was a non-specific, off-target effect of calpain inhibitors because we saw similar results with three different calpain inhibitors that act by different mechanisms. It is interesting to note that calpain activation seem to be play a key role in toxicity of CDDP in other cell and tissue types. For example, in CDDP-induced endothelial injury, there is calpain activation and calpain inhibitor PD-150606 is able to block endothelial cell apoptosis^21^ and calpain inhibitor MDL28170 is able to block cell death and tube breakdown in a 3-D culture of blood vessels^22^.

It is unclear if the rapid calpain activation we observe in CDDP-treated neurons in vitro is dependent on Sarm1 activation. Although we found that calpain activation in vivo was blocked in Sarm1^−/−^ mice, this is a late event and maybe due to blockade of Wallerian-like degeneration. It is however, curious that formation of DNA-platinum adducts in neurons was also partially blocked in the Sarm1^−/−^ mice, indicating that perhaps Sarm1 activation in CDDP treated neurons is a rapid event and that calpain activation downstream of Sarm1 may be partially responsible for the formation of DNA-platinum adduct formation.

Calpain activation likely plays a role in CDDP cytotoxicity. For example, in triple-negative breast cancer cells, activation of calpain 1 is required for CDDP-induced apoptosis^23^ and in human ovarian cancer cells, CDDP induces calpain activation and accumulation of mitochondrial and nuclear p53 by downregulating p53-associated parkin-like cytoplasmic protein^24^. However, in our experiments with three different cancer cell lines we did not see any effect of calpain activation on accumulation of DNA-platinum adduct formation, yet we clearly saw that DNA-platinum adduct formation was attenuated with different calpain inhibitors in neurons. Since Sarm1 expression is restricted to neurons and immune cells^25^, it is possible that the rapid calpain activation that is required for DNA-platinum adduct formation, and therefore neurotoxicity, with CDDP is dependent on Sarm1 activation and that selective expression of Sarm1 is responsible for the differential effects of Sarm1 in neurons versus cancer cells. Future experiments with selective Sarm1 inhibitors may be needed to examine the time course and temporal relationship between Sarm1 and calpain activation.

In summary, we have found that calpain activation is an early event in CDDP-induced neurotoxicity and formation of DNA-platinum adducts can be abrogated by calpain inhibition in neurons but not in cancer cells. We have also presented evidence that CDDP-induced axonal degeneration can be prevented by genetic deletion of Sarm1 gene. These findings suggest that Sarm1 inhibition to block the neurotoxicity of CDDP is unlikely to interfere with the anti-neoplastic effects of CDDP.

## Methods

All experiments involving animals were carried out in accordance with relevant guidelines and regulations and were approved by the Johns Hopkins Institutional Animal Care and Use Committee. Sources and catalogue numbers of relevant reagents are listed after each reagent below.

### Neurotoxicity assays

In order to examine neurotoxicity of CDDP in vitro*,* we used both a rat DRG neuronal cell line (50B11 cells) and primary embryonic rat DRG neuron-Schwann cell co-cultures as previously described^26,27^. 50B11 cells were plated to 96 well plates at a density of 3500 cells/well in Neurobasal medium supplemented with 1% Penicillin Streptomycin, 5% fetal bovine serum (FBS), 0.5 mM glutamine, B-27 supplement, 0.2% glucose for 24 h, then the cells were differentiated with 50 μM forskolin (Sigma-Aldrich, F6886) in culture medium with reduced serum (0.2%FBS). CDDP (Enzo, ALX-400-040-M050) was added at various concentrations and the cells were incubated for another 24 h. Toxicity was evaluated by the ViaLight Plus Cell Proliferation and Cytotoxicity Bioassay Kit (Lonza, LT07-121).

Primary rat DRG neuron-Schwann cell co-cultures are plated as described before^26^. Briefly, DRGs from Sprague‐Dawley rat embryos at 15th day of gestation were aseptically dissected and isolated. The cells were dissociated enzymatically with collagenase and trypsin and suspended in Neurobasal medium (Gibco, 21103‐049), with 1% fetal bovine serum (FBS) (Hyclone, 3H30071.3), 0.5 mM L‐Glutamine (Gibco, 25030‐081), 10 ng/ml glial cell line‐derived neurotrophic factor (GDNF, gift from Amgen), 2% B27 supplement (Gibco, 17504‐044), and 0.2% glucose. 96‐well sterile tissue culture plates were coated first with 100 µg/ml Poly‐D‐Lysine hydrobromide (Sigma‐Aldrich, P7280) and then with 10 µg/ml laminin (Invitrogen, 23017‐015). The DRG cells were cultured at a density of 6,000 cells/well, and incubated in a humidified 37 °C, 5% CO_2_ incubator. Their neurite growth and differentiation were visible under an inverted light microscope after 24 h of culture. CDDP was added to the wells at various concentrations and the cells were incubated for another 24 h. Toxicity was evaluated by the ViaLight Plus Cell Proliferation and Cytotoxicity Bioassay Kit (Lonza, LT07-121).

In order to evaluate axonal degeneration in primary DRG neurons, embryonic rat DRG neuron-Schwann cell co-cultures were established on collagen‐coated glass coverslips and incubated in media for 24 h to allow axons to grow. CDDP at various concentrations were added to the wells and the plates were incubated for another 24 h. Then, the cells were fixed with 4% paraformaldehyde and axons are stained with anti‐βIII‐tubulin antibody (Promega, G7121) according to standard immunostaining protocols. Vehicle media was added to serve as control in each plate. Longest axons of thirty randomly selected neurons were measured per well, and average axon length per well was calculated. Each experimental condition was done in triplicate wells and repeated several times to confirm reproducibility.

### In vitro protease assays

50B11 cells were plated in 96-well plates at a concentration of 6000 cells/well and incubated for 24 h, and differentiated in low serum media with forskolin as above. Untreated cells, vehicle control and assay media with no cells served as controls. CDDP at different concentrations were added to the wells and protease assays are performed every 4 h for a total of 48 h.

Proteasome-Glo (Promega, G8660) kit is used to assay for chymotrypsin-like proteasome activity, and Caspase-Glo 3/7 (Promega, G8091) kit is used for Caspase 3/7 activity in cultured cells. Both assays were carried out according to manufacturer’s protocols and the activity of each enzymatic assay that is proportional with luminescence was measured with a luminometer plate reader. Since Calpain-Glo (Promega, G8501) is designed for purified enzyme activity, we modified the original protocol to detect calpain activity in cells and tissues. The media in the wells were replaced with an ice-cold hypotonic buffer containing buffer (25 mM TrisHCl, 1 mM EDTA) and the cells were lysed with sonicator probe for 10 s and the cell lysates were added to the assay. As Suc-LLVY substrate is also a substrate for 20S proteasome, a selective 20S inhibitor, Epoxomicin (Millipore-Sigma, E3652) was added to the wells with a final concentration of 1 µM to block activity that is attributable to 20S proteasome and incubated 15 min in room temperature before calpain activity was measured with a luminometer plate reader. Calpain1 (Millipore, 208712), 20S proteasome (Enzo, BML-PW8720) and calpain inhibitor AK295 (Millipore Sigma, 208743) was used as positive and negative controls.

In order to examine whether calpain or caspase activation was upstream of each other, caspase and calpain activities were examined after application of specific inhibitors together with CDDP. 50B11 cells are plated in 96 well plates at a concentration of 6000 cells/well and incubated for 24 h, and differentiated in low serum media with forskolin as above. Untreated cells, vehicle control and assay media with no cells are served as controls. Calpain Inhibitor AK295 or pan-caspase inhibitor Z-VAD-FMK (Promega, G7231) were added at different concentrations together with 3 µM of CDDP to the wells and calpain and caspase activity were measured after 24 h as described above. All activities were normalized to protein concentration in each well.

### In vivo calpain activation

Eight-week old adult, male AJ mice (The Jackson Laboratory, Bar Harbor, ME) were given a single dose of CDDP (4 mg/kg) via tail vein injections. Sciatic nerves and DRGs were isolated at 1, 3, 5 and 7 days after the injections. Mice receiving saline for 7 days served as controls. The tissues were lysed with tissue homogenizer in an ice-cold hypotonic buffer containing 25 mM TrisHCl (pH 7.5), 1 mM EDTA and 1 µM Epoxomicin and calpain activity was measured using the above Calpain-Glo kit from Promega.

### In vivo model of cisplatin induced peripheral neuropathy

Sarm^−/−^ mice were obtained from a colony at the University of Massachusetts (courtesy of Dr. Marc Freeman) and bred in house. Genotyping was confirmed for each mouse using the protocols described in the original paper that generated the Sarm1 KO mouse^28^. Wild-type control C57BL/6 J mice were purchased from the Jackson Laboratory (Bar Harbor, ME). For both studies only, male mice aged 8–12 weeks were used.

Baseline thermal testing and electrophysiological evaluation were completed as described below and animals were randomly assigned to either CDDP or saline control groups. CDDP was dissolved freshly in sterile saline and administered at a dose of 4 mg/kg by tail vein injections once a week for 4 weeks to both Sarm1^−/−^ and wild type mice as previously described^29,30^. The injections were given at the same time each day using standard rodent injection techniques. Control groups received normal saline only. One week after the last dose of CDDP, nerve conduction studies and thermal sensation tests were done and mice were euthanized by isoflurane inhalation. Tissues were harvested for histopathological evaluation.

### Electrophysiological assessment

Baseline tests were carried one day before the start of injections and endpoint tests were done the day before the mice were euthanized for histopathological evaluation. Evoked sensory responses were recorded from the tail as previously described^30^. Briefly, mice were maintained under deep isofluorane anesthesia and recording electrodes were placed at the base of the tail and the stimulating electrodes were placed 5 cm distally. Orthodromic stimulation was carried and responses from 6 stimulations were averaged. Sensory nerve action potential (SNAP) amplitude and sensory nerve conduction velocity (SNCV) were calculated.

### Thermal sensation testing

Thermal sensation was evaluated using IITC Plantar Analgesia Meter 400 (Woodland Hills, CA) as described before^27,30^. For each group, measurements were made 2 days before the first injections and 2 days before euthanasia for the histopathological evaluation, at the end of the experiments. Mice were individually placed inside a containment box and allowed to acclimate to its surroundings for 15 min. The light source was then switched on, emitting heat onto the medial plantar surface of the subject’s left foot. The time taken for the mouse to lift this paw was measured 8 times with minimum 10-min intervals. 2 minimum and 2 maximum values are excluded for each animal and withdrawal latencies are averaged.

### Epidermal nerve fiber analysis

After the behavioral tests were done, animals were euthanized under deep inhalation anesthesia, and the footpads were harvested for the evaluation of intra epidermal nerve fibers (IENF). Two-mm punch biopsies of medial plantar footpads were fixed with paraformaldehyde-lysine-periodate fixative up to 24 h at 4 °C, kept in a cryoprotective solution, and cut serially with a freezing microtome with 50 µm thickness. The sections were stained with pan-axonal marker anti-protein gene product 9.5 (Bio-Rad, 7863–0504). For quantitative analysis, Intra epidermal nerve fibers were counted in 4–6 sections using a microscope at X40 magnification and IENF density calculated for each animal as described previously^27,31^ and averaged.

### Dot blot for DNA-platinum adduct identification

Examination of DNA-platinum adduct formation was done as described previously^30^. 50B11 cells were plated in 6-well plates at a concentration of 330,000 cells/well and incubated for 24 h. CDDP (3 µM) was added to the wells either alone or with caspase or calpain inhibitors at the following concentrations: pan-caspase inhibitor Z-VAD-FMK (50 µM), calpain inhibitors, AK295 (50 µM)^16^, Calpastatin (MilliporeSigma SCP0063) (5 µM), or MDL-28170 (100 µM)^28,29^. Untreated cells served as controls. The cells are incubated for 24 h and DNA was isolated using the DNeasy Blood and Tissue Kit according to the manufacturer’s protocol (Quanigen, 69504).

In order to compare the DNA-platinum adduct formation in neurons to cancer cells, cancer cell lines PA-1 (ATCC, CRL-1572) and Caov-3 (ATCC, HTB-75) were grown in DMEM with 10% FBS, the cancer cell line SK-OV-3 (ATCC, HTB-77) was grown in Mc Coy’s media with 10% FBS according to supplier’s protocols. 330,000 cells/well were plated into 6-well tissue culture plates and treated either with either 3 µM of CDDP alone or together with calpain inhibitors. Untreated cells are served as controls. After 24 h of incubation DNA was isolated using the DNeasy Blood and Tissue Kit according to the manufacturer’s protocol.

To evaluate the effects of CDDP on DNA-platinum adduct formation in Sarm1^−/−^ mice, CDDP or vehicle was administered at 4 mg/kg once a week through tail vein injection for 4 weeks to 8–12 weeks-old Sarm1^−/−^ or wild type mice. At the end of 4 weeks, lumbar DRGs and bilateral sciatic nerves were harvested and DNA was isolated using the DNeasy Blood and Tissue Kit according to the manufacturer’s protocol.

DNA isolated from the cells or tissues were denatured by adding tenfold volume 1 M NaOH and incubated at room temperature for 10 min^30^ and then blotted onto nylon membranes (Hybond-N; GE Healthcare, RPN203N) and allowed to dry. After crosslinking by UV light, membranes were incubated in blocking solution for 1 h. This was followed by incubation with anti-DNA/platinum adduct antibody (EMD-Millipore, MABE416) at 4 °C on shaker overnight. After washing the membrane 3 times for 10 min each, the membrane was incubated with secondary antibody (goat anti-rat IgG conjugated to HRP, (Millipore, AP136P) for 1 h at room temperature. The membrane was washed again 3 times (10 min each), developed with Immobilion Western Chemiluminescent HRP Substrate (Millipore, P90719) and exposed to X-ray film. For DNA loading control, membranes were stained with SYBR Gold Nucleic Acid Gel Stain (Thermofisher, S11494). ImageJ program was used to quantify dot blots.

## Supplementary Information

Supplementary Information.
