# Supplementary material for: Cisplatin induced neurotoxicity is mediated by Sarm1 and calpain activation
Source: Sci Rep. 2020 Dec 14;10:21889. doi: 10.1038/s41598-020-78896-w (PMC7736304; doi:10.1038/s41598-020-78896-w)
Supplement: Supplementary file 1 — Supplementary Information. [file 41598_2020_78896_MOESM1_ESM.pdf]

Supplementary Figures

Cisplatin induced neurotoxicity is mediated by Sarm1 and calpain activation

Aysel Cetinkaya-Fisgin, PhD, Xinghua Luan MD, PhD, Nicole Reed, MS, Ye Eun Jeong, MS, Byoung Chol Oh PhD, and Ahmet Hoke, MD, PhD

Correspondence: ahoke@jhmi.edu

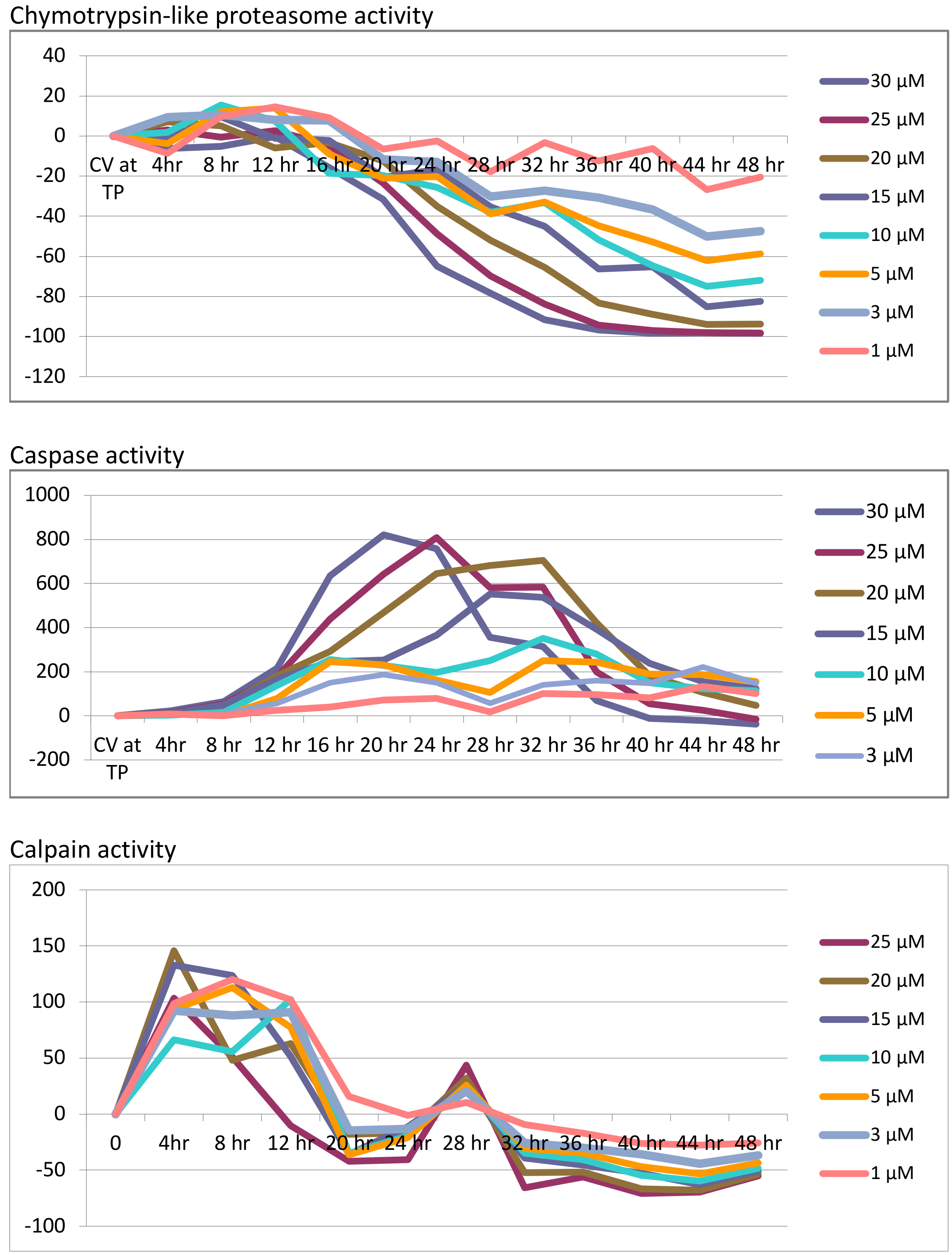

Supplementary Figure 1: CDDP inhibits the UPS and activates Caspase 3/7 and Calpains in a dose dependent manner. (n=4 at each time point)

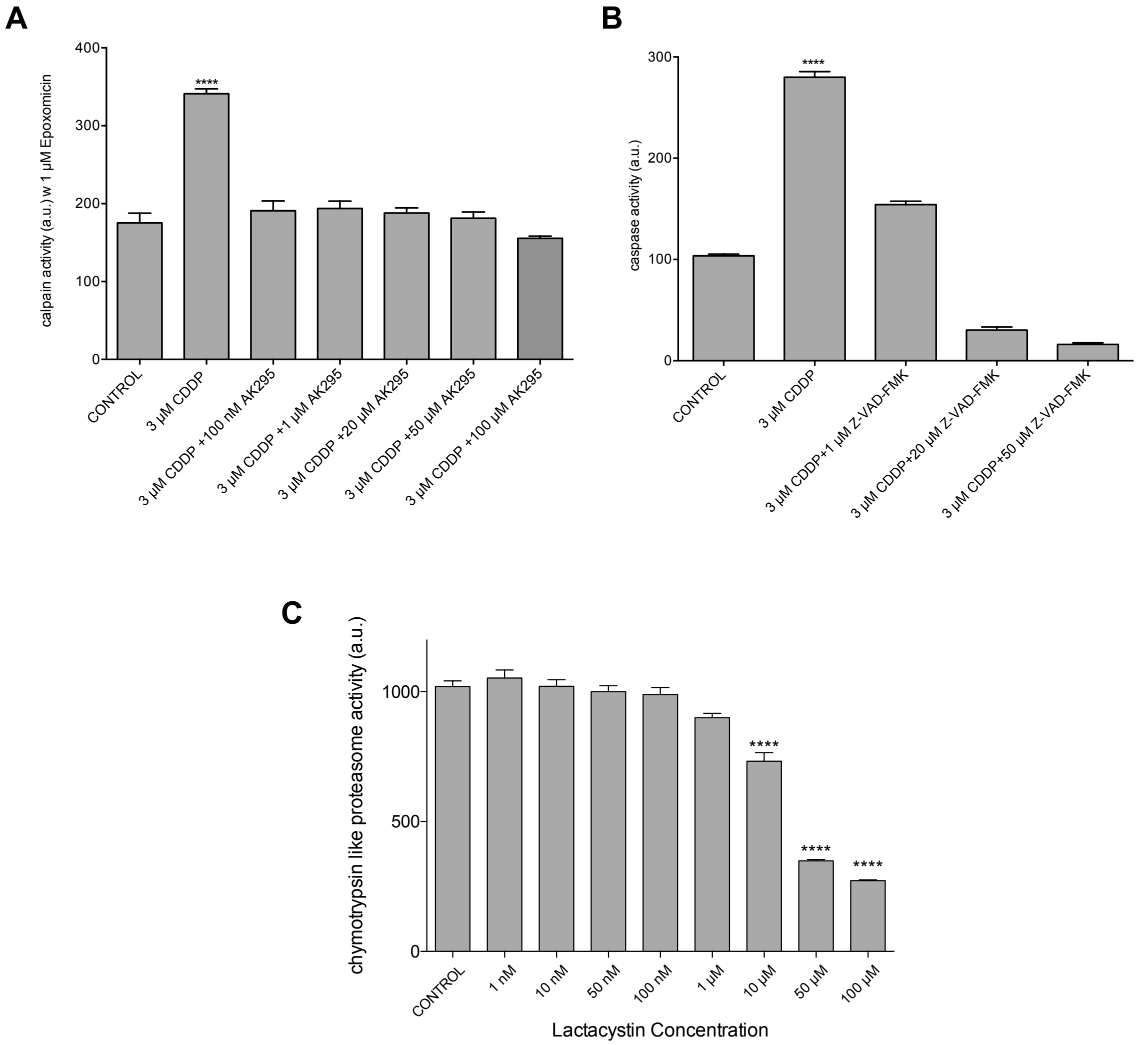

Supplementary Figure 2: The specificity of the Calpain-Glo and Caspase-Glo assays were evaluated with corresponding inhibitors (A and B) and inhibition of UPS by lactacystin was confirmed (C). Calpain activity was inhibited by AK295, a specific and highly potent inhibitor of calpain enzymatic activity (A). Similarly, caspase activity was inhibited by Z-VAD-fmk, a pan-caspase inhibitor (B). Lactacystin inhibited the UPS with a profile similar to that of CDDP.

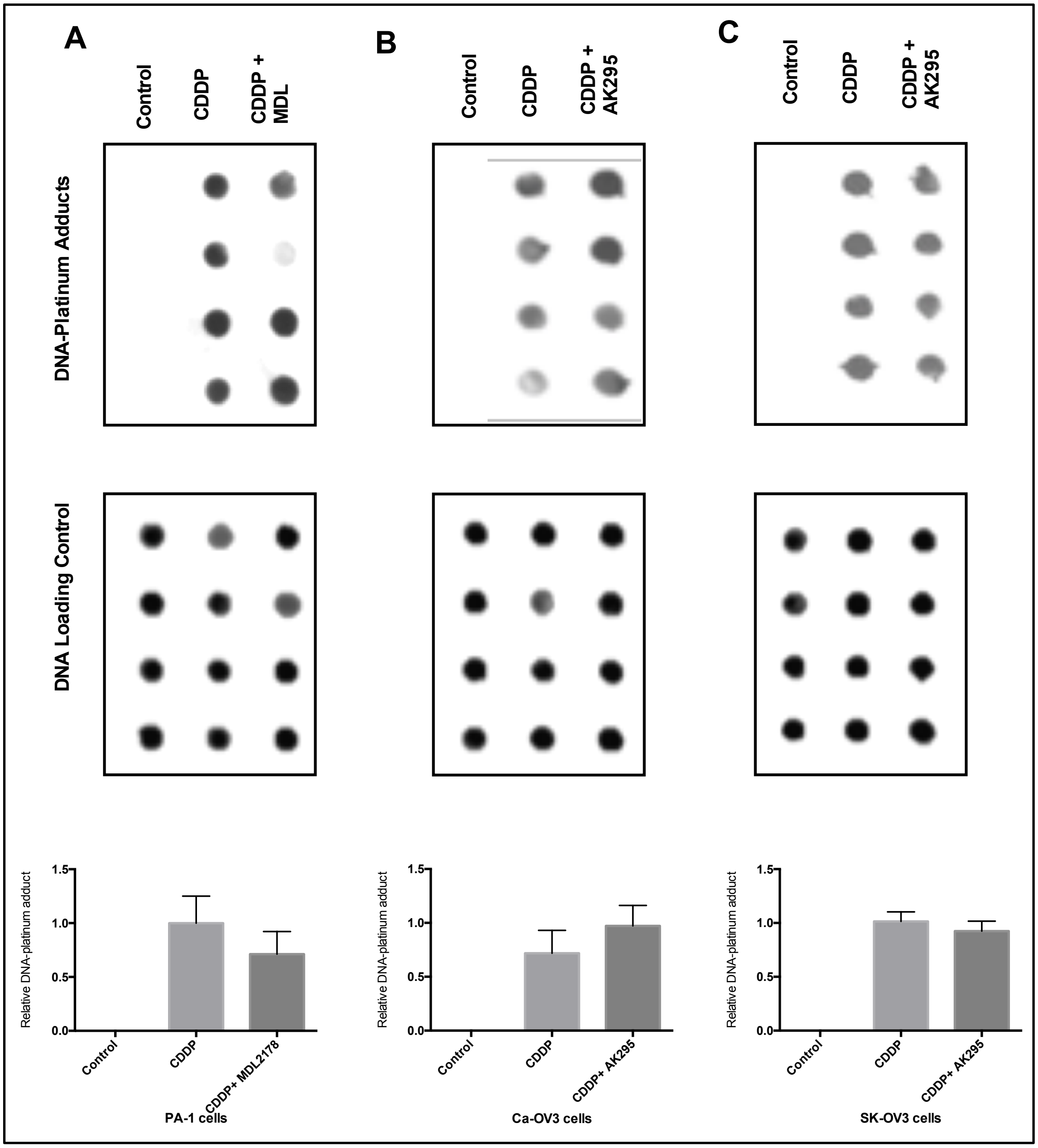

Supplementary Figure 3: Calpain inhibitors MDL2178 and AK295 do not affect DNA-Platinum adduct formation in three different cancer cell lines.
